# Multidomain intervention for dementia prevention: a scoping review

**DOI:** 10.3389/fneur.2026.1729290

**Published:** 2026-01-20

**Authors:** Xindi Guo, Chenhui Fan, Jiabao Ren, Ziyi Zhang, Cuiping Ni, Yu Liu

**Affiliations:** China Medical University Nursing School, Shenyang, China

**Keywords:** adaptive strategy, cognitive function, dementia, fingers, synergies

## Abstract

**Purpose:**

To map core components, outcomes, and challenges of multidomain dementia prevention interventions through a scoping review, and to compare differences in intervention effects between Chinese and non-Chinese countries/regions populations, thereby informing evidence-based strategies for localized interventions in China.

**Design:**

JBI-guided scoping review using Arksey and O’Malley’s framework.

**Data sources:**

Six databases (PubMed, Cochrane Library, Embase, Web of Science, CINAHL, PsycInfo) searched from inception to March 12, 2025.

**Methods:**

Peer-reviewed studies on multidomain interventions were screened via a two-stage process (title/abstract → full-text) using EndNote 21. Initial searches identified 2,968 articles, and 18 randomized controlled trials (RCTs) were finally included after duplicate removal and eligibility screening. Data extraction and synthesis followed.

**Results:**

Eighteen studies identified four core components of multidomain interventions: physical exercise, cognitive training, nutrition, and cardiovascular risk management. The more often reported implementation pattern was 3–5 weekly combined physical/cognitive sessions over 6–24 months. Regarding outcomes, cognitive function improvement, quality of life enhancement (+7.3 EQ-5D), and dementia risk reduction (40%) were reported in multiple studies, though inconsistent results existed. Subgroup analysis showed that Chinese studies had slightly lower cognitive improvement (MMSE +1.5–1.7) than Western studies (+1.8–2.1), but higher adherence (80% vs. 65% on average) due to family-participatory interventions. Key barriers included low adherence, resource limitations, and cultural disparities.

**Conclusion:**

Multidomain strategies are associated with addressing dementia risk factors, but existing evidence shows heterogeneity in intervention models and implementation barriers. Future research should focus on optimizing intervention models for Chinese populations, developing multidimensional assessments, and implementing culturally adaptive strategies to enhance scalability.

**Impact:**

These interventions are critical for dementia prevention in high-burden regions (e.g., China). Integrating evidence regarding differences between China and non-Chinese countries/regions into public health programs and tackling systemic barriers can enhance accessibility, equity, and feasibility, thus mitigating the societal impact of dementia amid global aging.

## 1 Introduction

Dementia represents the foremost public health challenge of the 21st century (1). With global population aging, the number of dementia patients and associated healthcare costs are rising rapidly (2). China bears the highest global burden of dementia, accounting for 25% of total cases worldwide (2, 3). Effectively reducing dementia prevalence is critical for achieving the “Healthy China” initiative. In 2024, the “Lancet Commission on Dementia Prevention, Intervention, and Care” updated 14 modifiable dementia risk factors, proposing that targeted interventions could prevent or delay up to 45% of global dementia cases (4). Given the multifactorial and complex etiology of dementia, multidomain interventions—integrating three or more independent lifestyle components—may serve as a pivotal strategy for optimal prevention (5). Evidence indicates that multidomain interventions significantly improve cognitive function and delay dementia progression compared to single-domain approaches (e.g., isolated physical or cognitive training), as they comprehensively address multiple risk factors to yield superior preventive outcomes (6). However, existing findings are predominantly derived from non-Chinese countries and regions, where disparities in dietary habits, physical fitness, and cultural contexts may restrict the direct applicability of these protocols in China. Thus, there is an urgent need to develop multidomain intervention strategies tailored to older Chinese populations.

This review was conducted according to the JBI guidance for scoping reviews (7) and is reported in accordance with the PRISMA extension for Scoping Reviews (PRISMA-ScR) (8). This study employs Arksey and O’Malley’s (9) scoping review framework to systematically search, collate, and synthesize global evidence on multidomain interventions for dementia prevention. We meticulously summarize intervention components, efficacy, and implementation barriers, aiming to provide a reference for future research in this field within China.

## 2 Data and methods

### 2.1 Defining research questions

Three research questions were formulated through preliminary literature review: (1) What core components are included in multidomain dementia-prevention interventions, and what are the distribution characteristics of their implementation frequencies? (2) What are the differences in outcome indicators between multidomain interventions and single-domain interventions? (3) What barriers are encountered during the implementation of multidomain interventions, and what coping strategies have been proposed in existing studies?

### 2.2 Literature search strategy

PubMed, Cochrane Library, Embase, Web of Science, CINAHL, and PsycInfo databases were systematically searched from inception to March 12, 2025. A combination of Medical Subject Headings (MeSH) and free-text terms was employed for the search. The PubMed search strategy is detailed in Table 1.

**Table 1:** PubMed database retrieval.

Steps	Retrieval type
#1	“Dementia”[MeSH]
#2	“Alzheimer Disease”[MeSH]
#3	“Cognitive Dysfunction”[MeSH]
#4	“Cognition Disorders”[MeSH]
#5	Cognitive Impairment [Title/Abstract] OR Cognitive Decline [Title/Abstract]
#6	#1 OR #2 OR #3 OR #4 OR #5
#7	Multidomain[Title/Abstract] OR Multicomponent[Title/Abstract] OR Multi-fields[Title/Abstract]
#8	“Methods”[MeSH]
#9	Intervention[Title/Abstract] OR Prevention[Title/Abstract] OR Training[Title/Abstract] OR Trial[Title/Abstract] OR Program[Title/Abstract] OR Change[Title/Abstract]
#10	#8 OR #9
#11	#6 AND #7 AND #10

#### 2.2.1 Note on grey literature retrieval

This study did not include grey literature (e.g., conference abstracts, government reports) because the inclusion criteria required “complete reporting of intervention components, frequencies, and outcome data,” while most grey literature lacks detailed methodological descriptions (e.g., unclear intervention duration, unreported sample size), making it difficult to meet the needs of core information extraction. Unpublished feasibility data can be supplemented in subsequent studies.

### 2.3 Inclusion and exclusion criteria

#### 2.3.1 Inclusion criteria

(1) Study design: Randomized controlled trials (RCTs); (2) Interventions: Comprising ≥3 independent multidomain components; (3) Participants: Healthy older adults or those at high risk of dementia, aged ≥60 years; (4) Intervention duration ≥6 months (10).

#### 2.3.2 Exclusion criteria

(1) Incomplete description of intervention protocols; (2) Duplicate publications; (3) Full text not retrievable after attempts to contact authors/libraries; (4) Non-English articles.

### 2.4 Literature screening and data extraction

Two researchers independently screened titles/abstracts and full texts and extracted data. Retrieved citations were imported into EndNote 21 to remove duplicates, followed by eligibility screening based on inclusion/exclusion criteria. Full texts of potentially eligible studies were reviewed. Discrepancies were resolved by a third researcher.

Data extraction was performed using a standardized Excel form, with cross-verification by a second researcher. Extracted data included: authors, country, study objectives, participant characteristics, intervention duration, multidomain components and frequencies, outcome measures, assessment tools, and implementation barriers.

**Figure 1:**
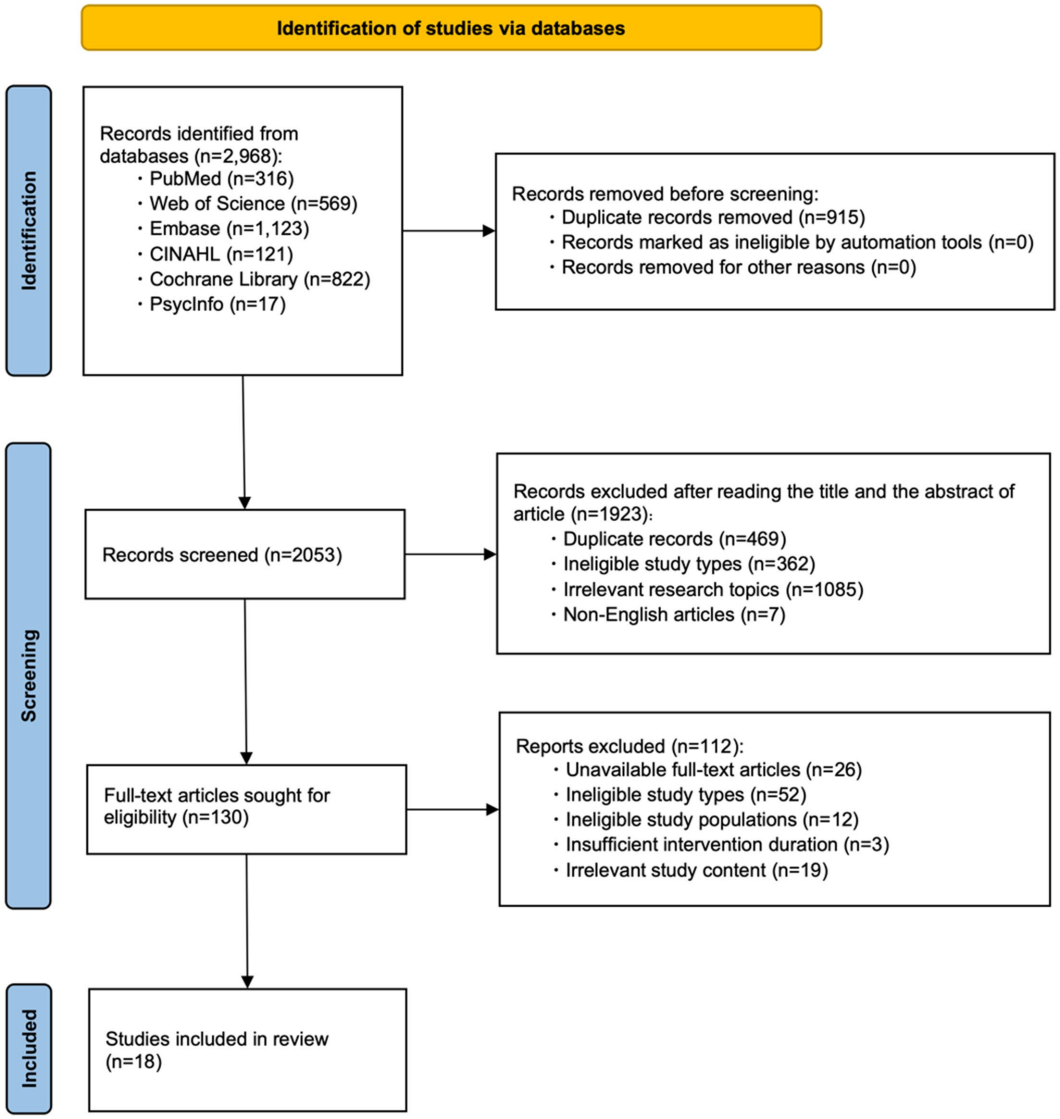
Literature screening process diagram.

A PRISMA 2020 flow diagram (adapted for scoping reviews) was used to document the screening process (Figure 1).

## 3 Results

### 3.1 Literature search results

Initial searches identified 2,968 articles, including PubMed (*n* = 316), Web of Science (*n* = 569), Embase (*n* = 1,123), CINAHL (*n* = 121), Cochrane Library (*n* = 822), and PsycInfo (*n* = 17). After removing 915 duplicates, 2,053 articles remained. A total of 1,923 articles were excluded after title/abstract screening (including 469 duplicates, 362 ineligible study types, 1,085 irrelevant research topics, and 7 non-English articles). Full texts of 130 potentially eligible articles were reviewed, and 112 were excluded (including 26 articles with unrecoverable full texts, 52 ineligible study types, 12 ineligible study populations, 3 with insufficient intervention duration, and 19 with irrelevant study content). Finally, 18 studies were ultimately included (11–28). The detailed screening flowchart is presented in Figure 1.

### 3.2 Characteristics of included studies

The 18 included studies originated from 11 countries: Finland (*n* = 2), France (*n* = 2), the Netherlands (*n* = 2), China (*n* = 2), Germany (*n* = 1), Singapore (*n* = 1), the United States (*n* = 3), South Korea (*n* = 3), the United Kingdom (*n* = 2), Spain (*n* = 1), and Latin America (*n* = 1). Key characteristics of the included studies are summarized in Table 2.

**Table 2:** Characteristics of the included studies (*n* = 18).

Author (Year)	Country/Region	Study population (age)	Duration (months)	Intervention components	Outcome measures (tools)	Implementation challenges
Ngandu et al. (11)	Finland	1,260 (60–77 years)	24	① ② ③ ④	A: Neuropsychological battery B: MMSE C: Zung Depression Scale D: SPPB	Long-term implementation monitoring difficulties Participant adherence Follow-up maintenance Complex outcome assessment
Moll van Charante et al. (12)	Netherlands	3,526 (70–78 years)	72	① ② ③ ④ ⑥ ⑦	A: MMSE and VAT C: GDS G: DSM-IV dementia criteria H: ALDS I: Record J: Record	Implementation quality assurance Resource constraints Data analysis limitations Ethical/regulatory issues
Wang et al. (13)	China	7,698 (>60 years)	Ongoing	① ② ③ ④	A: Cognitive composite D: Physical performance tests G: Follow-up H: Follow-up K: QoL Scale	Low diagnosis rates Resource limitations Stakeholder engagement Policy support gaps
Zülke et al. (14)	Germany	1,152 (60–77 years)	24	① ② ③ ⑥ ⑧	A: Cognitive composite C: GDS E: Barthel and IADL J: Record K: WHOQOL-OLD	Adherence challenges Technical/resource constraints Cultural barriers
Xu et al. (15)	Singapore	1,200 (66–70 years)	24	① ② ③ ④	A: Cognitive composite B: GDS E: ADL F: CDR K: HRQoL L: PSQI	Technical/resource constraints Cultural/language barriers
Pothier et al. (16)	France	120 (≥65 years)	6	① ② ③	A: Cognitive composite D: SPPB K: Euro-QoL and 5D-3L M: MNA and FFQ	Engagement challenges Technical implementation issues System reliability concerns
Baker et al. (17)	USA	2,000 (60–79 years)	24	① ② ③ ④	A: Cognitive composite C: GDS D: SPPB E: ADL F: CDR K: HRQoL L: PSQI	Technical/resource constraints Cultural barriers Stakeholder support gaps
Yaffe et al. (18)	USA	200 (70–89 years)	24	① ② ③ ④	A: Cognitive composite D: RAPA and ActiGraph K: PROMIS L: PSQI	Resource constraints Environmental interference
Park et al. (19)	Korea	150 (60–79 years)	6	① ② ③ ④ ⑨	A: MMSE and MoCA K: Euro-QoL and 5D-3L	Individual variability Cultural barriers Long-term evaluation gaps
Meng et al. (20)	China	96 (≥60 years)	6	① ② ③ ⑦ ⑧	A: Cognitive composite N: ULS-8 O: ANU-ADRI-SF	COVID-19 impacts Adherence/support challenges
Poppe et al. (21)	UK	704 (≥60 years)	24	① ② ③ ⑧ ⑩	A: Cognitive composite C: HADS D: Actigraphy K: EQ-5D-5L L: PSQI M: MEDAS N: BLS	Adherence issues Resource/cultural barriers
Essery et al. (22)	UK	360 (60–85 years)	12	① ② ③	A: Baddeley Reasoning Task E: IADL K: EQ-5D-5L	Adherence issues Technical/resource constraints Cultural barriers Implementation challenges Policy/social support gaps
Tainta et al. (23)	Spain	125 (>60 years)	12	① ② ③ ④	A: Cognitive composite C: GDS P: Record	Adherence issues Cultural barriers
Crivelli et al. (24)	Latin America	1,400 (60–77 years)	12	① ② ③ ④	A: Cognitive composite D: Record E: ADL K: EQ-5D M: Record P: Record	Cross-cultural collaboration difficulties Cultural/language barriers
Barbera et al. (25)	Finland France Netherlands	2,725 (≥65 years)	18	① ③ ④	A: CAIDE Dementia Risk Score C: Standardized depression tools D: CHAMPS Q: Framingham Risk Score	Adherence issues Long-term follow-up risks Technical diversity challenges
Tomaszewski et al. (26)	USA	225 (≥65 years)	6	① ② ⑧	A: Cognitive composite C: CES-D D: SPPB R: CSES	Adherence issues Technical/cultural barriers
Lee et al. (27)	Korea	460 (≥60 years)	18	① ② ③ ⑧ ⑩	A: MMSE C: GDS M: MDA	Adherence issues Long-term follow-up risks Technical/cultural barriers
Park et al. (28)	Korea	32 (≥60 years)	6	① ② ③ ④ ⑧	B: CERAD-NB D: SPPB E: IADL K: EQ-5D O: ANU-ADRI	Adherence issues Resource/cultural constraints

① Exercise. ② Cognition. ③ Nutrition. ④ CV Monitoring. ⑤ Omega-3. ⑥ Medication. ⑦ Education. ⑧ Socialization. ⑨ Motivation. ⑩ Behavior.

A-Cognition. B-Global cognition. C-Depression. D-Physical function. E-Activities of daily living. F-Dementia severity. G-Incidence. H-Disability. I-Cardiovascular events. J-Mortality. K-Quality of life. L-Sleep. M-Nutrition. N-Loneliness. O-Dementia risk. P-Adherence. Q-Cardiovascular risk. R-Self-efficacy.

### 3.3 Core components and optimal frequencies of multidomain interventions

Multidomain interventions have been widely applied in dementia prevention research. The 18 included studies identified four core components: physical exercise, cognitive training, nutritional guidance, and cardiovascular risk factor management.

Physical exercise (100% of studies) primarily included aerobic exercise, strength training, and balance training, delivered via group or individualized programs. The more often reported was 3–5 sessions per week (20–60 min/session). The FINGER trial (11) demonstrated that high-intensity exercise (5 sessions/week) significantly improved cognitive function. Cognitive training (94.4% of studies) employed computer-based programs targeting memory, executive function, and other domains, typically delivered 2–3 times weekly (15–30 min/session). Nutritional guidance (88.9% of studies) focused on Mediterranean diet recommendations, Omega-3 supplementation, and personalized dietary advice. Monthly consultations (1–2 sessions/month) showed efficacy, though Omega-3 adherence was lower in Latin American populations (24). Cardiovascular risk management (77.8% of studies) involved regular blood pressure and metabolic parameter monitoring. The eMIND trial (16) highlighted the feasibility of remote monitoring technologies.

The overall intervention duration ranged from 6 months to 2 years. Ngandu et al. (11) reported optimal outcomes with ≥3 weekly sessions combining physical and cognitive training over 2 years.

For detailed information on the specific intervention objectives, component combinations, and implementation frequencies of each included study, refer to the Appendix. This table systematically extracts the core intervention-related data from Table 2, thereby providing a specific basis for direct comparison of multidomain intervention protocols across different regions and study designs.

### 3.4 Outcomes of multidomain interventions and differences from single-domain interventions

The 18 included studies evaluated 18 outcome measures, covering cognitive function, quality of life, dementia risk, and physical function. Specifically, 83.3% of the studies reported outcomes related to cognitive function. Tools used to assess cognitive function included the Mini-Mental State Examination (MMSE) (29), neuropsychological battery (30), and Montreal Cognitive Assessment (MoCA) (31), among others. Among the included studies, 11 articles (11, 12, 14–18, 20, 21, 24, 25) evaluated the cognitive function of participants at three time points, namely baseline, mid-intervention, and post-intervention, while the remaining 6 articles (13, 19, 22, 23, 26, 27) only assessed cognitive function at baseline and post-intervention endpoint. In addition, 5 studies (11, 12, 22, 24, 26) conducted regular follow-up after the end of the intervention to observe the long-term effects of multidomain interventions on cognitive function. Moreover, the results of all the above-mentioned articles indicated a significant improvement in cognitive function. Additionally, 55.6% of studies showed improvements in quality of life, as measured by the EuroQol Five-Dimensional Descriptive System (EQ-5D) (32), with an average increase of 7.3 points. Long-term interventions lasting at least 2 years were associated with a 40% reduction in risk of developing dementia (33).

Multidomain interventions were significantly more effective than single-domain interventions in slowing cognitive decline. For example, the multidomain FINGER trial (11) reported a cognitive composite score improvement of +0.21 standard deviations (SD), which was significantly higher than the +0.08 SD observed in the single-domain EXERT trial (34). However, direct head-to-head comparisons were lacking, and differences may also be attributed to intervention frequency and participant characteristics.

### 3.5 Implementation barriers and coping strategies

Multidomain interventions commonly face barriers such as low participant adherence (18, 20), resource limitations (16, 20), and cultural disparities (14, 20).

Low adherence manifests as failure to adhere to intervention protocols or complete follow-ups, attributable to factors such as forgetfulness (12), lack of motivation (13), side effects (14, 23), or intervention complexity (19). Strategies to improve adherence include setting personalized goals (21) and providing material incentives. Crivelli et al. (24) highlighted that adherence varies across cultural contexts, emphasizing the need to consider cultural influences on participant engagement. Resource constraints, including shortages of healthcare professionals (12–15, 18–20, 28), limited facilities (12, 13, 15, 16, 19–24), recruitment challenges (14, 23), and funding limitations (15, 17), restrict the scope and depth of interventions. These issues can be mitigated through the use of digital technologies for remote monitoring (16) and community-based collaborative models (13). Cultural differences also impact intervention efficacy. Crivelli et al. (24) observed cultural diversity across Latin American countries and adapted interventions by substituting local staples (e.g., corn/beans) to enhance acceptability.

Furthermore, as multidomain interventions are implemented over the long term, dropout rates tend to increase. Tainta et al. (23) addressed this by shortening session durations, while Wang et al. (13) promoted family involvement to strengthen support systems, thereby enhancing the long-term sustainability of multidomain interventions.

### 3.6 Subgroup analysis of intervention effects among populations from China and non-Chinese countries/regions

Two Chinese studies (13, 20) and 16 studies from non-Chinese countries/regions were included in the subgroup analysis, with key differences as follows:

#### 3.6.1 Cognitive improvement

Chinese studies showed MMSE increases of 1.5–1.7 points, slightly lower than studies from non-Chinese countries/regions (1.8–2.1 points) (11–17). This discrepancy may be attributed to differences in intervention frequency—Chinese studies typically adopted 1–2 sessions/week of moderate-intensity exercise [e.g., Tai Chi in Meng et al. (20)], while studies from non-Chinese countries/regions commonly used 3–5 sessions/week of high-intensity exercise [e.g., jogging in Ngandu et al. (11) and Baker et al. (17)].

#### 3.6.2 Adherence

Chinese studies achieved significantly higher adherence rates (80–82%) compared to studies from non-Chinese countries/regions (58–72%) (18–21). This advantage was mainly due to the integration of family-participatory intervention models [e.g., rural family support in Wang et al. (13), where family members assisted with intervention adherence monitoring] and cultural emphasis on group compliance. In contrast, studies from non-Chinese countries/regions [e.g., Yaffe et al. (18) and Poppe et al. (21)] reported lower adherence primarily due to insufficient individual motivation, despite the use of material incentives [e.g., vouchers in Poppe et al. (21)].

#### 3.6.3 Barriers and adaptations

The main barrier in Chinese studies was low digital device penetration in rural areas (13), addressed through paper-based cognitive games and community health worker home visits to avoid relying on digital tools. In studies from non-Chinese countries/regions, the primary barrier was insufficient individual motivation (18) and low cultural acceptability of dietary components [e.g., 45% Omega-3 adherence in Latin American studies due to mismatched dietary habits, Crivelli et al. (24)]. Strategies from non-Chinese countries/regions to address these issues included personalized feedback and localized dietary adjustments [e.g., replacing Nordic fish with local staples in Crivelli et al. (24)].

## 4 Discussion

### 4.1 Diverse forms of multidomain interventions: exploring optimal models

Multidomain interventions encompass various components, including nutritional guidance, physical exercise, cognitive training, and cardiovascular risk factor monitoring and management. These interventions demonstrate significantly superior efficacy in improving cognitive function and preventing dementia compared to single-domain approaches. Studies have shown that single-domain interventions, such as the Mediterranean diet (35), aerobic and strength training (34), and cognitive training targeting memory, reasoning, and processing speed (36), can yield positive effects.

However, given the multifactorial and complex etiology of dementia, multidomain interventions achieve optimal outcomes through synergistic targeting of multiple pathways: the Mediterranean diet inhibits *β*-amyloid deposition (37), physical exercise enhances hippocampal neurogenesis and cerebral blood flow (38), cognitive training activates prefrontal-parietal network connectivity (39), and cardiovascular risk management reduces the risk of vascular dementia (4). These combined effects comprehensively address multiple risk factors, resulting in superior preventive efficacy.

The effectiveness and sustainability of multidomain interventions are influenced by implementation models. For example, Ngandu et al. (11) demonstrated that high-frequency interventions (3–5 sessions/week) significantly outperformed the low-frequency model employed by Yaffe et al. (18) in improving cognitive and physical function. Therefore, future research should investigate the relationship between intervention dosage and outcomes to define the optimal multidomain intervention model.

### 4.2 Complex efficacy of multidomain interventions: necessity of multidimensional evaluation systems

The complexity of multidomain interventions necessitates efficacy evaluations encompassing physiological, psychological, and social functional dimensions. Among the 18 included studies, a total of 18 outcome measures were examined; however, 83.3% relied on a single metric for assessment. For instance, the SUPERBRAIN trial (19) and GOIZ ZAINDU trial (23) evaluated cognitive function solely using the MMSE, while the U.S. POINTER trial (17) and APPLE TREE trial (21) adopted a multidimensional approach integrating biomarkers, mental health, and quality of life.

Although standalone cognitive tests (e.g., MMSE or MoCA) can assess cognitive improvements, they fail to capture the holistic effects of multidomain interventions on physiological parameters, mental health, and social functioning (4). Neuroimaging data and blood biomarkers (e.g., plasma Aβ42/Aβ40 ratio) can elucidate mechanisms underlying neurodegenerative inhibition (40). The U.S. POINTER trial (17) demonstrated that multidomain interventions significantly reduced Alzheimer’s disease pathological burden through Aβ42/Aβ40 analysis. Mental health and social impacts can be evaluated using tools such as the Geriatric Depression Scale (GDS) (41), EQ-5D, and social participation questionnaires. The APPLE TREE trial (21) revealed a significant positive correlation between quality of life improvements and family support intensity in the intervention group via multidimensional assessments.

Therefore, future studies should adopt multidimensional evaluation systems, integrating core metrics [e.g., MMSE/MoCA for cognition, ADL/IADL for daily functioning (42, 43), Aβ/inflammatory biomarkers for physiology (44, 45)] with extended assessments (e.g., GDS/HADS for mental health, social function questionnaires, and qualitative interviews) to comprehensively evaluate the efficacy of multidomain interventions.

### 4.3 Multidomain interventions face multiple barriers: adaptive strategies are required

Multidomain interventions encounter multiple barriers during implementation, including low participant adherence, resource constraints, and cultural disparities. Adaptive strategies must be developed to ensure intervention feasibility and sustainability.

Low adherence is a pervasive challenge (44). For instance, in the SMARRT trial (18), 28% of participants withdrew due to insufficient motivation or health status changes. Adherence issues may arise from intervention complexity, lack of immediate feedback, or health fluctuations (46). Strategies to enhance adherence include simplifying protocols, setting personalized goals, and offering material incentives (6). For example, the GOIZ ZAINDU trial (23) increased completion rates to 89% by reducing session duration from 60 to 30 min. The APPLE TREE trial (21) achieved higher adherence in the intervention group (72% vs. 55% in controls) through personalized goal-setting, feedback, and £20 vouchers.

Resource constraints—such as shortages of healthcare professionals, inadequate facilities, and limited funding (47)—hinder intervention scalability. These challenges can be addressed via digital alternatives and leveraging existing community resources (48). The eMIND trial (16) reduced staffing needs by 50% and costs by 30% using digital health technologies for remote cognitive training and monitoring. Similarly, the MIND-China trial (13) minimized facility costs by collaborating with rural and community health centers.

Cultural differences also affect intervention acceptability (49). The LATAM-FINGERS trial (24) addressed dietary disparities in Latin America by substituting Nordic fish with local staples (e.g., corn/beans), increasing participant acceptance from 45 to 82%. Thus, multidomain interventions must be culturally adapted to local dietary habits, educational levels, and sociocultural contexts to enhance feasibility.

### 4.4 Evidence gaps identified by this scoping review

This review identified three key evidence gaps:

Insufficient evidence on Chinese rural populations: Only 1 study (13) focused on rural China, and no studies explored interventions for low-income or ethnic minority groups, limiting the generalizability of findings to diverse Chinese populations.

Unclear dose–response relationship: The association between intervention frequency/duration and outcomes remains unclear, and no studies have determined the “minimum effective dose” for Chinese populations, making it difficult to balance intervention effectiveness and feasibility.

Limited long-term follow-up data: Most Chinese studies have a duration of ≤6 months, and long-term outcomes (e.g., dementia incidence) are lacking, making it impossible to evaluate the sustainability of intervention effects.

## 5 Conclusion

This scoping review systematically mapped global evidence on multidomain dementia prevention interventions, identifying four core components (physical exercise, cognitive training, nutrition, cardiovascular risk management) and their distribution characteristics in implementation frequencies. Multidomain interventions have been associated with improvements in cognitive function, quality of life, and reduction in dementia risk, but evidence inconsistencies and regional differences exist. Subgroup analysis showed that Chinese studies have higher adherence but slightly lower cognitive improvement than studies from non-Chinese countries/regions, mainly due to differences in intervention frequency and cultural adaptation strategies.

The primary contribution of this review is to highlight evidence gaps relevant to Chinese populations, including insufficient rural evidence, unclear dose–response relationships, and limited long-term data. Future research should: Develop localized intervention models for Chinese rural and low-income populations, integrating family participation and community resources. Explore the dose–response relationship of interventions to determine the optimal balance between effectiveness and feasibility. Conduct long-term follow-up studies to evaluate the sustainability of intervention effects. Establish multidimensional evaluation systems to comprehensively assess intervention impacts on cognitive, physical, and social function.

This review also has limitations: it only included RCTs and excluded grey literature, which may have missed unpublished feasibility data; there was insufficient age-stratified data in the included studies, and the impact of baseline status on intervention effects was overlooked, so future studies need to design dual-dimensional stratified intervention trials based on “age-cognitive risk”; the included studies adopted inconsistent definitions of high dementia risk and heterogeneous assessment tools for the same outcome indicators, which may impair the comparability between studies; almost none of the included studies reported dementia incidence after intervention completion, which may hinder cross-study comparisons; and there was insufficient reporting of long-term follow-up outcomes in the included studies, with no analysis of the long-term effects of multidomain interventions; and due to the nature of scoping reviews, no quantitative synthesis of outcomes was conducted. Future systematic reviews with meta-analysis can further quantify intervention effects and explore moderating factors.

## Data Availability

The original contributions presented in the study are included in the article/Supplementary material, further inquiries can be directed to the corresponding author.
