# Supplementary material for: Multidomain intervention for dementia prevention: a scoping review
Source: Front Neurol. 2026 Jan 20;17:1729290. doi: 10.3389/fneur.2026.1729290 (PMC12864128; doi:10.3389/fneur.2026.1729290)
Supplement: Supplementary file 1 [file Table_1.docx]

| Author (Year) | Primary Objective | Intervention Components & Frequency |
| --- | --- | --- |
| Ngandu et al.^[11]^ (2015) | Evaluate multidomain interventions' effectiveness in preventing cognitive decline among elderly at risk | ① 3-5 sessions/week (20-60 min/session) ② Biweekly group education (2hrs/session)+Individual training（5 sessions/week , 15 min/session) ③ 3 individual + 7-9 group consultations ④ Quarterly consultations |
| Moll et al.^[12]^ (2016) | Assess cardiovascular health interventions' effectiveness in dementia prevention for community-dwelling elders | ①-④⑥ Quarterly consultations ⑦ 5 sessions during study |
| Wang et al.^[13]^ (2022) | Establish baseline data and test interventions to understand rural Chinese elders' health status and dementia prevention strategies | ①-④ Customized frequency (weekly/monthly) |
| Zülke et al.^[14]^ (2019) | Prevent/delay cognitive decline in German high-risk elders through multidomain interventions | ① 3-5 sessions/week (20-30 min) ② 3 sessions/week (15 min) ③⑥⑧ Personalized schedule |
| Xu et al.^[15]^ (2022) | Evaluate effectiveness and safety of interventions for Singaporean high-risk elders | ① 1-3 sessions/week (20-60 min) ② 3 sessions/week (15-30 min) ③ 3 individual + 6 group sessions ④ Quarterly/semiannual tests |
| Pothier et al.^[16]^ (2018) | Assess web-based interventions' feasibility and acceptability for dementia prevention | ① ≥2 sessions/week (30 min) ② ≥2 sessions/week (15 min) ③ 2 sessions/month (5-8 min) |
| Baker et al.^[17]^ (2024) | Compare efficacy of two multidomain intervention intensities for dementia risk reduction with cultural adaptation and community sustainability evaluation in U.S. settings | ① 2-4 sessions/week (15-35 min) ② ≥3 sessions/week (30 min) ③④ Regular group meetings |
| Yaffe et al.^[18]^ (2019) | Assess personalized multidomain strategies' impact on elders' cognition and dementia risk profiles | ①-④ Individualized guidance every 4-6 weeks |
| Park et al.^[19]^ (2020) | Evaluate feasibility and adherence of multidomain cognitive/brain health interventions for Korean elders with modifiable dementia risks | ① 3 sessions/week (60 min) ② 2 sessions/week (50 min) ③ 7 group + 3 individual sessions ④ Monthly ⑨ 4 group consultations(50 min) |
| Meng et al.^[20]^ (2024) | Implement life-course model multidomain interventions to reduce dementia risk and enhance cognition in Chinese high-risk elders, establishing evidence-based prevention strategies | ① Monthly  ② ≥3 sessions/week (15 min) ③⑦ Weekly ⑧ Monthly |
| Poppe et al.^[21]^ (2022) | Evaluate clinical effectiveness of multidomain interventions for cognitive decline reduction in high-risk elders | ①-③⑧⑩ Biweekly group meetings (1hr) |
| Essery et al.^[22]^ (2022) | Evaluate multidomain interventions' feasibility/acceptability for cognitive health maintenance in high-risk populations | ①-③ ≥1 website visit/week |
| Tainta et al.^[23]^ (2024) | Adapt and evaluate the FINGER multidomain dementia prevention methodology for older adults in Southern Europe (Basque region | ① 2 group + ≥150min individual/week ② 20hrs group + 3 individual(20 min)/week ③ 2 group meetings ④ Weekly/monthly |
| Crivelli et al.^[24]^ (2023) | Validate FINGER trial efficacy in Latin America while exploring multidomain interventions' cognitive protection potential and advancing cross-cultural dementia research equity | ①②③④Customized weekly/monthly schedule |
| Barbera et al.^[25]^ (2018) | Implement web-based multidomain interventions to optimize cardiovascular risk profiles in older adults ≥65y, preventing cardiovascular/cognitive decline and dementia | ①③④On-demand platform access |
| Tomaszewski et al.^[26]^ (2023) | Develop digital app-supported interventions integrating memory training and lifestyle modifications to mitigate cognitive decline in high-risk elders | ①②⑧The intervention course will be conducted once a week for the first 10 sessions, once every 2 weeks thereafter, and once a month for the last 3 months, lasting for 6 months. |
| Lee et al.^[27]^ (2014) | Assess cognitive impacts of multidomain lifestyle interventions in community elders to identify effective decline-delaying strategies | ①②③⑧⑩Provide phone management or face-to-face consultation once every two months |
| Park et al.^[28]^ (2019) | Evaluate efficacy of multidomain lifestyle interventions in reducing dementia risk among high-risk elderly populations | ①②③④⑧Weekly group + monthly individual sessions |
| ① Exercise ② Cognition ③ Nutrition ④ CV Monitoring ⑤ Omega-3 ⑥ Medication ⑦ Education ⑧Socialization ⑨ Motivation ⑩ Behavior | | |
